# Developmental Changes in Sensory-Evoked Optical Intrinsic Signals in the Rat Barrel Cortex

**DOI:** 10.3389/fncel.2017.00392

**Published:** 2017-12-12

**Authors:** Mikhail Sintsov, Dmitrii Suchkov, Rustem Khazipov, Marat Minlebaev

**Affiliations:** ^1^Laboratory of Neurobiology, Kazan Federal University, Kazan, Russia; ^2^INMED-INSERM U901, Aix-Marseille University, Marseille, France

**Keywords:** OIS, barrel field, development, neonatal, rats, light scattering, transparency, hemodynamic response

## Abstract

Optical Intrinsic Signal imaging (OISi) is a powerful technique for optical brain studies. OIS mainly reflects the hemodynamic response (HR) and metabolism, but it may also involve changes in tissue light scattering (LS) caused by transient cellular swelling in the active tissue. Here, we explored the developmental features of sensory-evoked OIS in the rat barrel cortex during the first 3 months after birth. Multispectral OISi revealed that two temporally distinct components contribute to the neonatal OIS: an early phase of LS followed by a late phase of HR. The contribution of LS to the early response was also evidenced by an increase in light transmission through the active barrel. The early OIS phase correlated in time and amplitude with the sensory-evoked electrophysiological response. Application of the Modified Beer-Lambert Law (MBLL) to the OIS data revealed that HR during the early phase involved only a slight decrease in blood oxygenation without any change in blood volume. In contrast, HR during the late phase manifested an adult-like increase in blood volume and oxygenation. During development, the peak time of the delayed HR progressively shortened with age, nearly reaching the stimulus onset and overlapping with the early LS phase by the fourth postnatal week. Thus, LS contributes to the sensory-evoked OIS in the barrel cortex of rats at all ages, and it dominates the early OIS phase in neonatal rats due to delayed HR. Our results are also consistent with the delayed blood oxygen level dependent (BOLD) signal in human preterm infants.

## 1. Introduction

Optical Intrinsic Signal imaging (OISi) is a simple yet powerful technique of minimal invasiveness for studying brain activity. It has been successfully used for studies of sensory functional topography (Grinvald et al., 1986; Bonhoeffer and Grinvald, 1991; Rubin and Katz, 1999; Kalatsky et al., 2005) as well as cerebral metabolism and hemodynamics (Malonek and Grinvald, 1996; Jones et al., 2001; Sheth et al., 2004). In clinical studies, functional Near-Infrared Spectroscopy (fNIRS), a completely noninvasive implementation of optical brain imaging, has become a routine technique for brain studies (Wyatt et al., 1986; Villringer et al., 1993; Obrig, 2014). In contrast to adult OIS, HR-based neuroimaging in neonates may be less reliable. Indeed, HR in the neonatal brain is characterized by weaker and slower responses (Arichi et al., 2012; Allievi et al., 2016). Additionally, poor neurovascular coupling (NVC) and immaturity of cerebral vascular autoregulation (Greisen, 2005; Harris et al., 2011) may result in an “anomalous” HR even in healthy neonates, making the interpretation of HR-based neuroimaging difficult. Indeed, while neuronal activity in adults evokes a positive HR, in neonates, it may produce a negative or biphasic HR (Born et al., 2000; Yamada et al., 2000; Kozberg et al., 2013) and a sensory-evoked decrease in cerebral blood flow (CBF) (Zehendner et al., 2013). Considering the high demand for optical techniques in clinical studies of human infants, development of robust recording methods and clear interpretation of the neonatal optical signals based on HR and other activity markers is of great importance.

In adults, fNIRS and OISi are both considered to be effective substitutes for functional Magnetic Resonance Imaging (fMRI) (Strangman et al., 2002; Huppert et al., 2006; Cui et al., 2011), even though the techniques differ considerably in resolution, dimensionality, and intrinsic activity markers. However, a wide range of markers other than HR may contribute to optical signals, comprising an enlarged feature space for optical studies. The optical feature space includes different chromophores (hemoglobin, cytochrome, water) (Jobsis, 1977; Grinvald et al., 1986; Matcher et al., 1994), *NADH*/*FADH*_2_ fluorescence (Mayevsky and Chance, 1982; Mayevsky and Rogatsky, 2007), tissue light scattering (LS) and transmission (MacVicar and Hochman, 1991; Aitken et al., 1999). In addition, optical studies are preferable to fMRI for human infants, especially for extremely preterm infants, due to easier light penetration through the thinner skull and scalp, bed-side setup portability and measurement feasibility (Greisen et al., 2011). However, to interpret optical imaging results, it is important to understand the mechanisms underlying OIS responses in the developing brain.

In the present study, we aimed to characterize sensory-evoked OIS in the somatosensory cortex of neonatal rats during their development using multispectral OISi and concomitant intracortical recordings of the electrophysiological signals. Newborn rat pups served as a convenient animal model of preterm human infants (Workman et al., 2013). Our main finding is that OIS in neonatal rats consists of two temporally distinct phases: an early decrease in the tissue light scattering (LS) followed by a delayed positive HR. During development, the HR delay shortens and largely overlaps with LS by the fourth week of postnatal development. Also, in neonatal rats, the early LS component functionally correlates in time and amplitude with the sensory-evoked neuronal activity.

## 2. Materials and methods

### 2.1. Surgery

All animal-use protocols followed the guidelines of the French National Institute of Health and Medical Research (INSERM, provisional approval N007.08.01) and the Kazan Federal University on the use of laboratory animals (ethical approval by the Institutional Animal Care and Use Committee of Kazan State Medical University N9-2013). Wistar rats of both sexes from postnatal days [P] 2–25 and 60–90 were used (P0 was the day of birth). The surgery was performed under isoflurane anesthesia (5% for induction and 1.5% during surgery). The rat skull was cleaned of skin and periosteum and was covered with dental cement (Grip Cement) except for a 5 × 5 mm^2^ window above the barrel cortex. The metal plate attached to the cement helmet was mounted to the ball joint in a stereotaxic apparatus. Subsequently, the rats were warmed and left for an hour to recover from anesthesia, surrounded by a cotton nest and heated via a thermal pad (35–37°C). All the recordings were made under urethane anesthesia (by intraperitoneal injection; 1 g/kg). To remove visual artifacts skull thinning or a simple skull polishing was performed depending on animal age. The skull was then covered with saline and a coverslip.

### 2.2. OIS recordings and analysis

**Figure 1:**
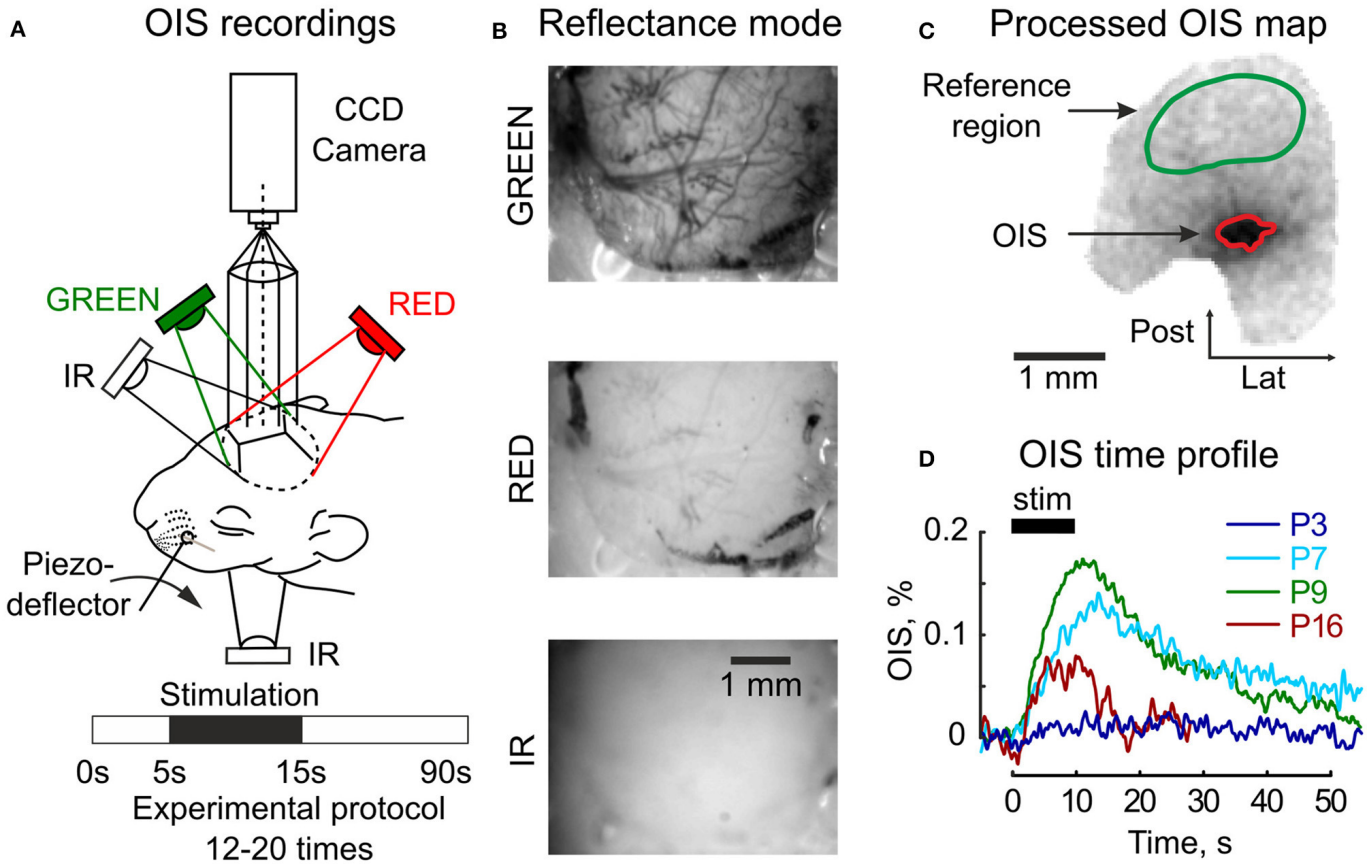
Setup design for the OIS recordings. **(A)** Three diodes (GREEN, RED, IR) are used for the reflectance mode imaging and one (IR) diode under the head—for the transmission mode imaging. The experimental protocol consists of 5 s of baseline registration, 10 s of tactile whisker stimulation, 45 s of recovery recordings, and an additional 30 s are reserved to store data. **(B)** Raw frames recorded in the reflectance mode in three imaging spectra (GREEN, RED, and IR) in the somatosensory cortex of a P6 rat. **(C)** An example of an OIS map recorded in the somatosensory cortex of a P6 rat in the reflected RED light; a green mask depicts the reference region used for the illumination correction and a red mask—the OIS region used to calculate a time course of the response. **(D)** Examples of the OIS time courses recorded in the reflected RED light in four animals of different ages (P3, P7, P9, P16). Each trace shows an average of 12–20 individual trials.

Multispectral OIS was recorded using a video acquisition system (Figure 1A). A CCD camera (QICAM Fast 1394) was positioned above the barrel cortex located by stereotaxic coordinates (Khazipov et al., 2015). The camera was focused at 200–1,200 μ m below the skull, approximately the depth of the L4 of the barrel cortex as indicated by electrophysiological activity (Mitrukhina et al., 2015). The cortex was illuminated by light diodes synchronized with the camera by an Arduino Uno microcontroller. Video frames were recorded at 130^*^174 resolution (1px = 35 μ m) at a frame rate of 5 Hz for each diode. Two imaging modes were used: reflectance and transmission. In the reflectance mode, the diodes were positioned above the skull around the head to produce spatially uniform illumination. In the transmission mode, only one IR diode was installed below the head facing toward the camera.

Multispectral OISi was performed using diodes with different wavelengths to reflect volumetric and oximetric changes of HR as described in Hillman (2008); Kozberg et al. (2013). Tissue light scattering (LS) changes were assessed with an additional IR diode. Two sets of experiments were performed independently with two different diode sets: (I) 525 nm and a diode modified to 626 nm and (II) 574 and 610 nm (Table S1, Figure S1). To increase the experimental sample size, the results of the two sets were then pooled together. The modified 626 nm diode was built from a 630 nm 3W diode (Arlight) and three film filters SG86, E703, E5455 (Rosco Roscolux and Supergel filter pack) in order to match the effective hemoglobin extinction coefficients of 610 nm. The actual spectra of the diodes were measured by a Thorlabs CCS175 spectrometer. To calculate the averaged extinction coefficients of oxy- and deoxyhemoglobin (ϵ*HbO* and ϵ*HbR*), the spectrophotometric data of rat blood absorption was used (Zijlstra et al., 1994). Based on our calculation of hemoglobin extinction coefficients we may conclude that OIS in the GREEN spectrum mainly reflects blood volumetric changes, in the RED spectrum—mainly blood oximetric changes and in the IR spectrum—mainly tissue transparency changes. However, to facilitate diode notations, they are referred to only by their spectral ranges: GREEN, RED, and IR. Example images of the neonatal cortex in the reflectance mode for each spectrum are shown in Figure 1B.

Natural sensory stimulation was performed as described previously (Peterson and Goldreich, 1994; Borgdorff et al., 2007). A single vibrissa was deflected by trains of brief pulses (10 ms pulse duration, 10 s train duration, 90 s inter-train interval, repeated 12–20 times) using piezo deflectors (Noliac) triggered by a pulse stimulator (A.M.P.I. Master8). OIS amplitude in the barrel cortex is known to depend on stimulation frequency in an age-dependent manner (Sheth et al., 2005), therefore to evoke the strongest response the whisker deflection rate was set at the optimal frequency depending on animal age (see Results).

Data preprocessing included per-frame spatial filtering with 2D Gaussian filter (σ = 2*px*, 1*px* = 35 μm) and a subsequent illumination artifact correction. The illumination correction consisted of, first, calculating the reference time profile averaged over the reference region *I*_*ref*_(*t*) and, second, subtracting it from each pixel intensity *I*(*t*): *I*_*corr*_(*t*) = *I*(*t*) − α*I*_*ref*_(*t*), where α – the normalization coefficient: α = 〈*I*(*t*)/*I*_*ref*_(*t*)〉_*t*_. The preprocessed frames were then averaged across all trials (*N*_*trials*_ = 12 − 20). The experimental protocol was one-conditioned, so the OIS map was calculated using the first-frame subtraction approach as in (Grinvald et al., 1986): *OIS* = (*I*_*corr*_(*stim*) − *I*_*corr*_(*baseline*))/*I*(*baseline*), where *I*(*stim*) and *I*(*baseline*) are intensities averaged within stimulation and baseline periods respectively. Active tissue was detected by an operator as darkening in the OIS map (Figure 1C). To calculate OIS time profile, the intensities of pixels within the OIS region were averaged. To facilitate visualization of OIS in reflectance mode, the signals were inverted (Figure 1D).

### 2.3. LFP recordings and analysis

Local field potential (LFP) was recorded using either a silicon probe (NeuraNexus A1x16-5mm-100-413-A16) or a glass pipette filled with artificial cerebrospinal fluid (resistance of 2–6 MOhm). The electrode was inserted into L4 of the principal barrel at a 30° angle to the skull surface which allowed simultaneous recordings of OIS. LFP signals were then amplified and digitized by DigitalLynx (Neuralynx) at 10 kHz. To measure the neuronal activity associated with OIS, LFP was averaged over the stimulation period.

## 3. Results

### 3.1. OIS development profile

We performed single whisker-evoked OISi in the barrel cortex in *N* = 120 rats aging from P2 to P90 (P0—the day of birth). An imaging experiment was considered to be successful if OIS was found in at least one of the spectra. We separated the experiments into 8 groups according to the rat ages (success rates in parentheses): P2-3 (1 of 8), P4-5 (8 of 19), P6-7 (24 of 47), P8-10 (10 of 16), P11-14 (4 of 5), P15-18 (6 of 8), P21-25 (4 of 13), P60-90 (3 of 4). The success rate in the P2-3 group was not high enough, and this group was excluded from further analysis. Nevertheless, an example of a successful OIS profile in a P3 rat is shown in Figure 1D.

OIS is typically characterized by a low signal-to-noise ratio (SNR). To increase the dynamic range of OIS recordings and to evoke the strongest signal, we first optimized the stimulation protocol. To do so, we modulated stimulation frequency from 0.1 Hz (1 stimulus) to 20 Hz (200 stimuli) and calculated the OIS amplitude recorded in the RED spectrum. Stimulation intensity is known to affect OIS amplitude in a nonlinear fashion (Sheth et al., 2004, 2005). We referred to the stimulation as being optimal if it evoked the strongest OIS compared to the other stimulation protocols. We found that the nonlinear relationship between stimulation and OIS amplitude persisted in early development. However, the optimal stimulation frequency increased with animal maturation (Figure S2). Due to the low SNR of optical signals, we analyzed the 3 OIS with the highest amplitude and referred to their stimulation rates as optimal. We found that the optimal stimulation frequency was 1 Hz during the first week and that it increased with age to attain 10 Hz by the fourth postnatal week. The Pearson correlation coefficient between stimulation rates and centers of group ages was of 0.65. For further imaging, we chose the following optimal stimulation rates: 1 Hz for groups P4-5 and P6-7, 2 Hz—for P8-10, 5 Hz— for P11-14 and 10 Hz—for P15-18 and older.

**Figure 2:**
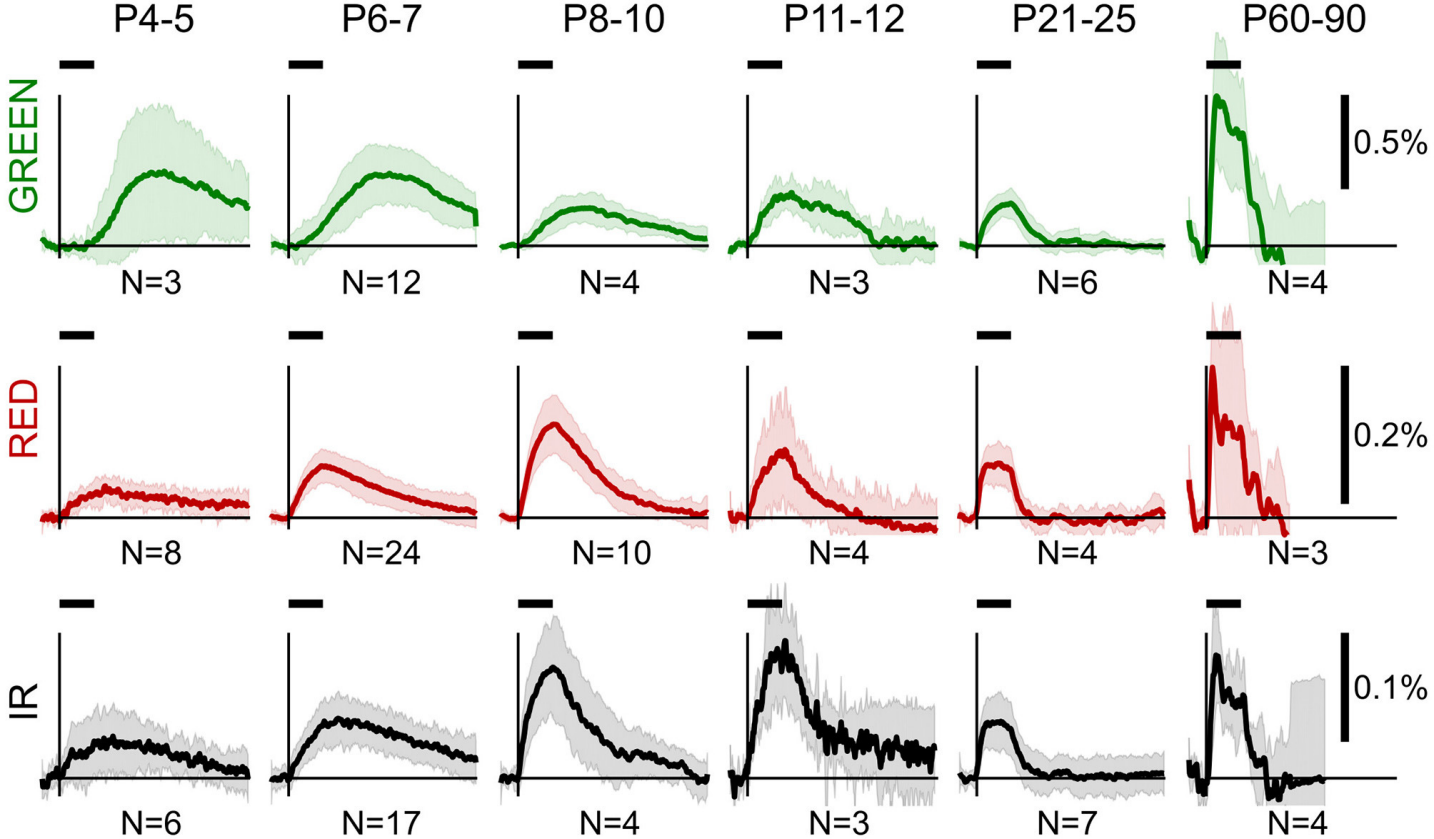
Group average OIS in different spectra at different ages. Plots show average OIS recorded in the reflectance mode in different age groups (P4-5, P6-7, P8-10, P11-12, P21-25, P60-90) and different imaging spectra (GREEN, RED, and IR). A solid line depicts a group average and a shaded region—a group's standard deviation of individual OIS traces. Vertical lines indicate the stimulus onset and a horizontal bar above indicates the stimulation period. The group size is shown below each plot.

Group data of OIS profiles obtained in the reflectance mode are shown in Figure 2. From a visual inspection of the group data, we observed several general features of OIS during development. Firstly, sensory-evoked OISs were slow in the neonatal rats, but their dynamics accelerated with age: the older the rat—the closer the OIS peak to the stimulation onset. Secondly, the time course of the RED signal closely resembled the IR signal in neonates. However, the IR signal was typically smaller than the RED one. Finally, neonatal OISs in different spectra were segregated in time, e.g., in the P6-7 group the GREEN signal peaked after the RED and the IR signals did.

**Figure 3:**
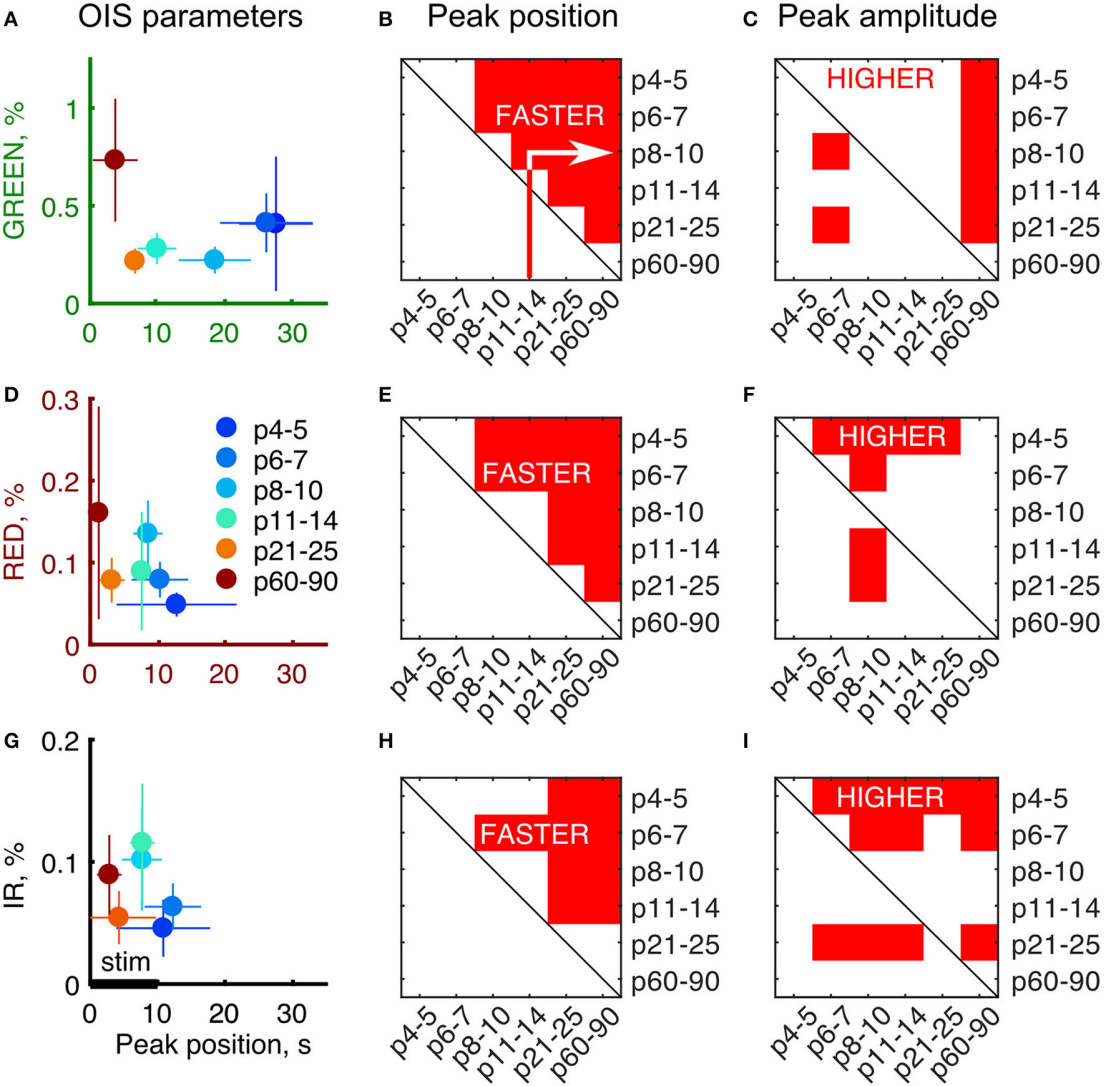
Developmental profile of the OIS parameters. **(A,D,G)** Peak amplitudes vs. peak positions plot for each of the imaging spectra. Circle markers represent a group average and error lines—a group's standard deviation. Number of animals as in Figure 2. **(B,E,H)** Results of the one-tailed Mann-Whitney U-test for peak positions for each of the group pair combinations. Red areas are the conditions when peak positions in the X axis are significantly smaller than the ones in the Y axis (*P* ≤ 0.05) meaning that OIS develops faster in X rather than in Y. **(C,F,I)** Results of the one-tailed Mann-Whitney U-test for peak amplitudes for each of group combinations similarly to **(B,E,H)**. Red areas are the conditions when peak amplitudes in the X axis are significantly higher than the ones in the Y axis (*P* ≤ 0.05) meaning that OIS is higher in X rather than in Y.

To quantitatively assess the developmental changes in OIS we calculated OIS peak amplitude and OIS peak position—a time delay from the stimulation onset to a peak (Figures 3A,D,G). We calculated an amplitude as the 95-percentile threshold of the signal distribution. The peak position was calculated as the first time sample where OIS reached its peak amplitude counting from the stimulation onset. Statistical significance of the difference between groups was assessed using a one-tailed Mann-Whitney U-test to find at which age OIS develops faster and attains a higher amplitude (*P*-value threshold was set at 0.05). We found that the peak position exhibited a monotonic decline with age for all spectra, e.g., OIS in the P11-14 group always grew faster than that in the P6-7 group (Figures 3B,E,H). The peak amplitudes, in contrast, displayed a nonlinear developmental profile (Figures 3C,F,I). In the GREEN spectrum, in the P8-10, P11-14, and P21-25 groups OIS amplitudes were significantly lower than those in the P6-7 or P60-90 groups, in the RED spectrum the P8-10 group exhibited a significantly higher amplitude than those of the P4-5, P6-7, P11-14 or P21-25 groups. It is noteworthy that signals in the RED and IR spectra shared similar parameters in neonates. Indeed, in the P6-7 group OIS peaked at 10.5 ± 4.0 s for RED and 12.5 ± 4.0 s for IR while the peak amplitude was of 0.08 ± 0.02% for RED and 0.06 ± 0.02% for IR.

The OIS peaks in the GREEN spectrum in the P4-5 (27.5 ± 5.5 s), P6-7 (26.0 ± 7.0 s) and P8-10 (18.5 ± 5.5 s) groups were attained significantly later than the 10 s stimulation train offset (*P* < 0.05, one-tailed Sign test). Considering that the GREEN spectrum imaging reflects mainly blood volumetric changes which, in turn, are correlated with CBF (Grubb et al., 1974), our data suggest that functional hyperemia in these groups is significantly delayed from the stimulation. However, in the other spectra, RED and IR, we observed that the OIS peak was attained within the stimulation period starting in the P8-10 group (8.5 ± 2.0 s in RED and 7.5 ± 3.0s in IR, *P* < 0.05, one-tailed Sign test) and in all the groups older than that.

### 3.2. Transmission and reflectance imaging modes in neonatal rats

**Figure 4:**
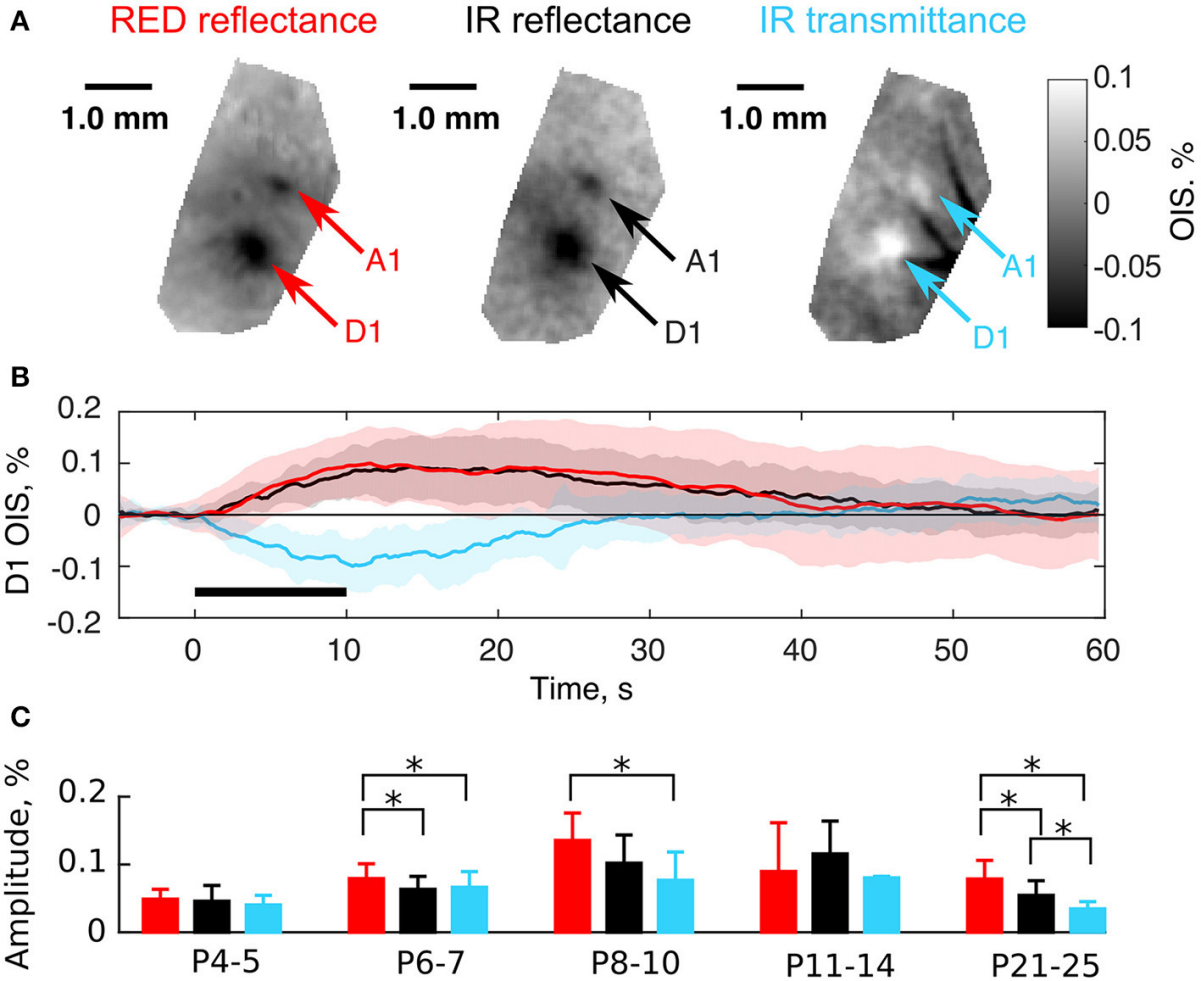
Comparison of the transmission and the reflectance imaging modes in the neonatal rats. **(A)** Examples of OIS maps recorded in a P6 rat using RED reflected, IR reflected, and IR transmitted light. Arrows indicate OIS regions corresponding to the A1 and D1 barrels. **(B)** OIS time courses averaged in the D1 OIS region of **(A)**. Solid lines represent trial-averaged OIS and the shaded region—standard deviation (*N*_*trials*_ = 20). The black horizontal line depicts stimulation interval. Note that OISs are inverted in polarity (upwardly-directed signals correspond to darkening and downwardly-directed signals correspond to brightening). **(C)** Group statistics of OIS absolute amplitudes recorded in the reflectance and the transmission modes in different age groups. A bar height equals to a group average and error lines—to a group's standard deviation. Each group consists of at least three animals. The asterisk (^*^) represents significant differences in group amplitudes (Mann-Whitney U-test, *P* < 0.05).

As mentioned above, OIS in the RED and IR spectra shared similar parameters in neonates (Figure 3). We hypothesized that this similarity was accounted for by LS changes in the nervous tissue. The light scattering coefficient has a nearly uniform spectrum in the visible and near-infrared regions (van der Zee et al., 1993; Ma et al., 2016) in comparison with the hemoglobin spectrum (Zijlstra et al., 1994). To test this hypothesis we compared OIS in transmission and reflectance modes, as it is known, that a scattering-affected OIS inverts, when moved from the reflectance to the transmission imaging mode (Aitken et al., 1999). We observed that in contrast to adult rats, a neonatal rat's head is highly transparent to near-infrared light due to its smaller size and thinner bones, but it is barely transparent to visible light. We compared OIS in RED reflected, IR reflected, and IR transmitted modes. The results of imaging in these modes in a P6 rat are shown in Figures 4A,B. Interestingly, in the transmission mode, sensory-evoked OIS appeared as a brightening of the sensory-stimulated cortical region contrary to the darkening in the same region in the reflectance mode (frame by frame comparison in Supplementary Video 1). Also, the dynamics of transmitted OIS closely matched the reflected OIS in neonatal rats. We calculated absolute values of amplitudes and found, that in the P4-5 group the amplitudes differences between imaging modes was nonsignificant (0.05 ± 0.01% for RED refl., 0.05 ± 0.02% for IR refl. and 0.04 ± 0.01% for IR trans.), however by the fourth week all imaging modes significantly differed from each other (0.08 ± 0.03% for RED refl., 0.05 ± 0.02% for IR refl. and 0.03 ± 0.01% for IR trans., Mann-Whitney U-test, *P* < 0.05) (Figure 4C). The increase in the activated tissue transparency manifested as brightening in the transmittance mode OIS, and darkening in the reflectance mode OIS, indicates a considerable contribution of LS to the neonatal OIS.

### 3.3. Early scattering response in neonatal rats correlates with the neuronal activity

**Figure 5:**
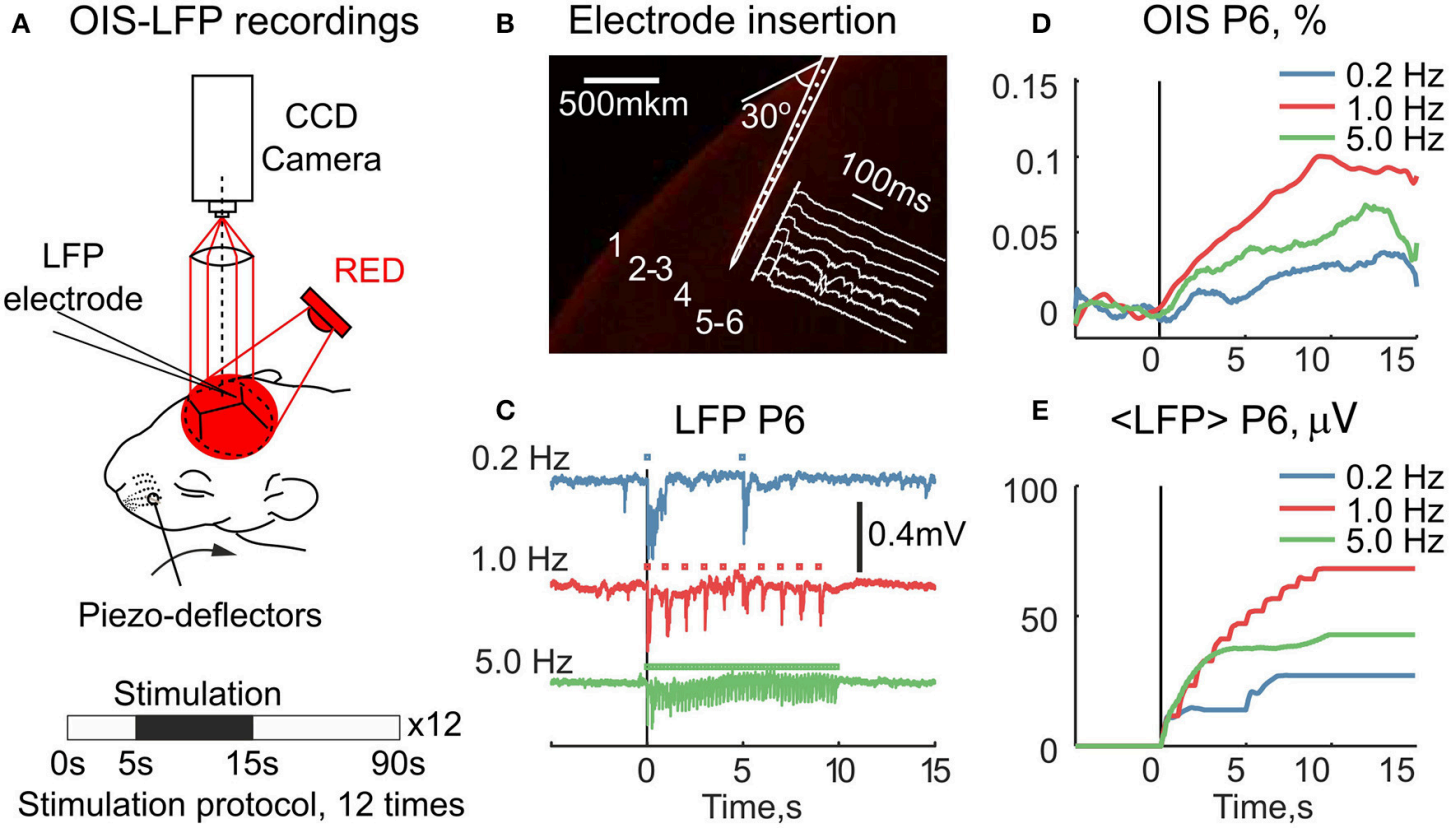
Simultaneous recordings of the RED OIS and electrical activity in the barrel cortex of neonatal rats. **(A)** Setup for simultaneous registration of OIS and LFP. **(B)** An example of a silicon probe inserted into the principal barrel of a P6 rat. The probe was stained with DiI (Sigma, 42364) before insertion. **(C)** LFP traces at different stimulation rates. Square markers indicate individual stimuli within the train. **(D,E)** The time course of the simultaneously recorded RED OIS and average LFP (< *LFP* >) in a P6 rat.

We further asked how the early OIS phase correlates with the electrophysiological response in neonatal rats. For this, we simultaneously recorded sensory-evoked LFP and OIS in the RED spectrum in P5-7 rats (*N*_*rats*_ = 4) (Figure 5A). A 16-channel silicon probe was inserted into the principal barrel, which was first located by OISi. To avoid interference with OIS recordings the probe was inserted at a 30° angle to the skull surface (Figure 5B). For the neuronal activity analysis, only the channel with the strongest evoked LFP response was used. To vary the amount of the neuronal activity, we modulated the stimulation frequency. To quantify the responses we averaged cumulative LFP over the stimulation period. Examples of raw LFP and its average are shown in Figures 5C,E. The corresponding OIS is shown in Figure 5D.

**Figure 6:**
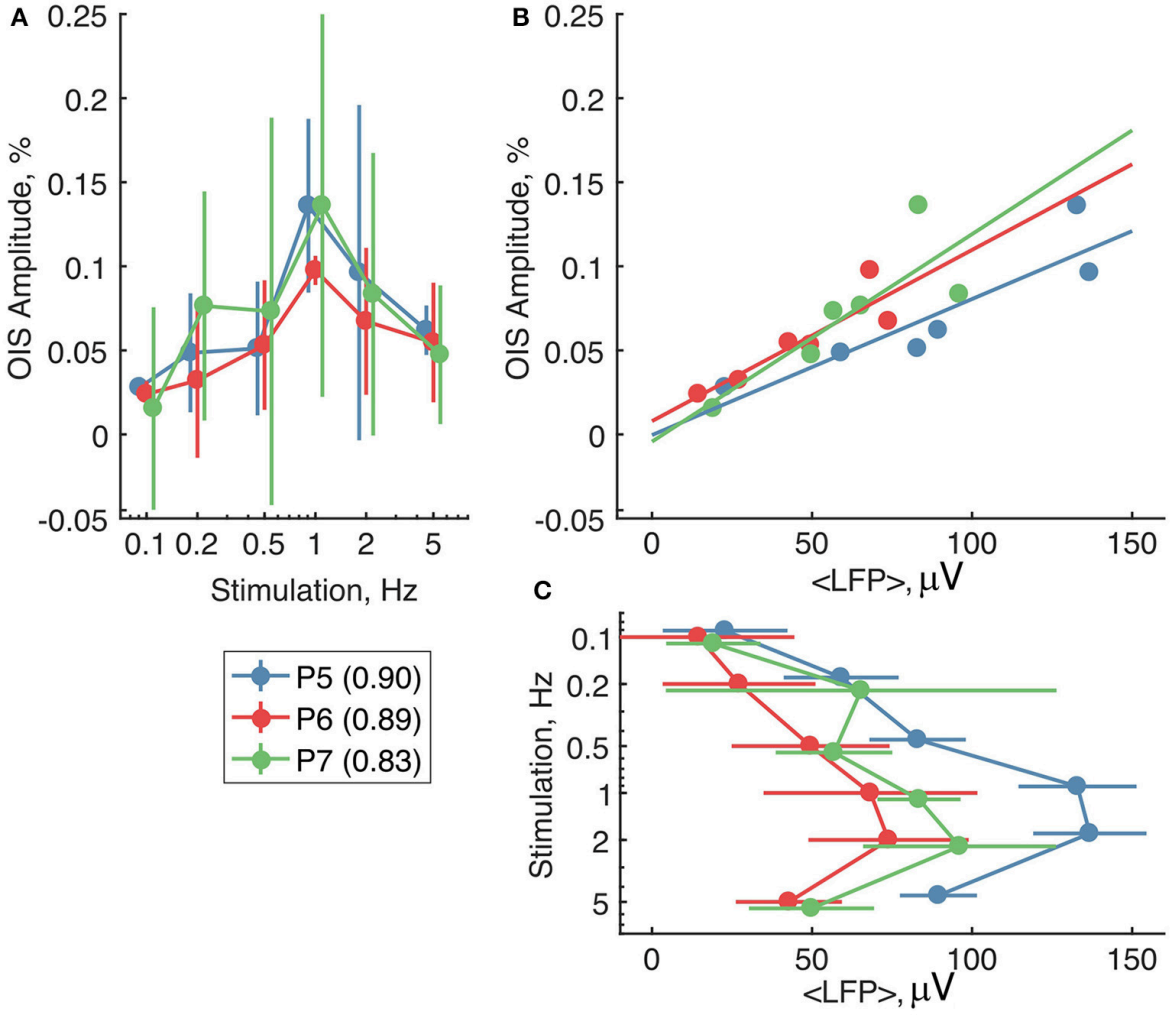
The relationship between the RED OIS amplitude, average LFP (< *LFP* >), and stimulation frequency in the neonatal rats (P5-P7). **(A,C)** Dependence of the RED OIS amplitude and average LFP on stimulation rate. **(B)** The relationship between the RED OIS amplitude and average LFP. Circle markers represent group average and error lines—a group's standard deviation of values in trials (*N*_*trials*_ = 12). The solid lines on **(B)** represent a linear regression of OIS amplitude vs. average LFP. Values of Pearson correlation coefficients for each experiment are shown in parentheses in the legend.

**Figure 7:**
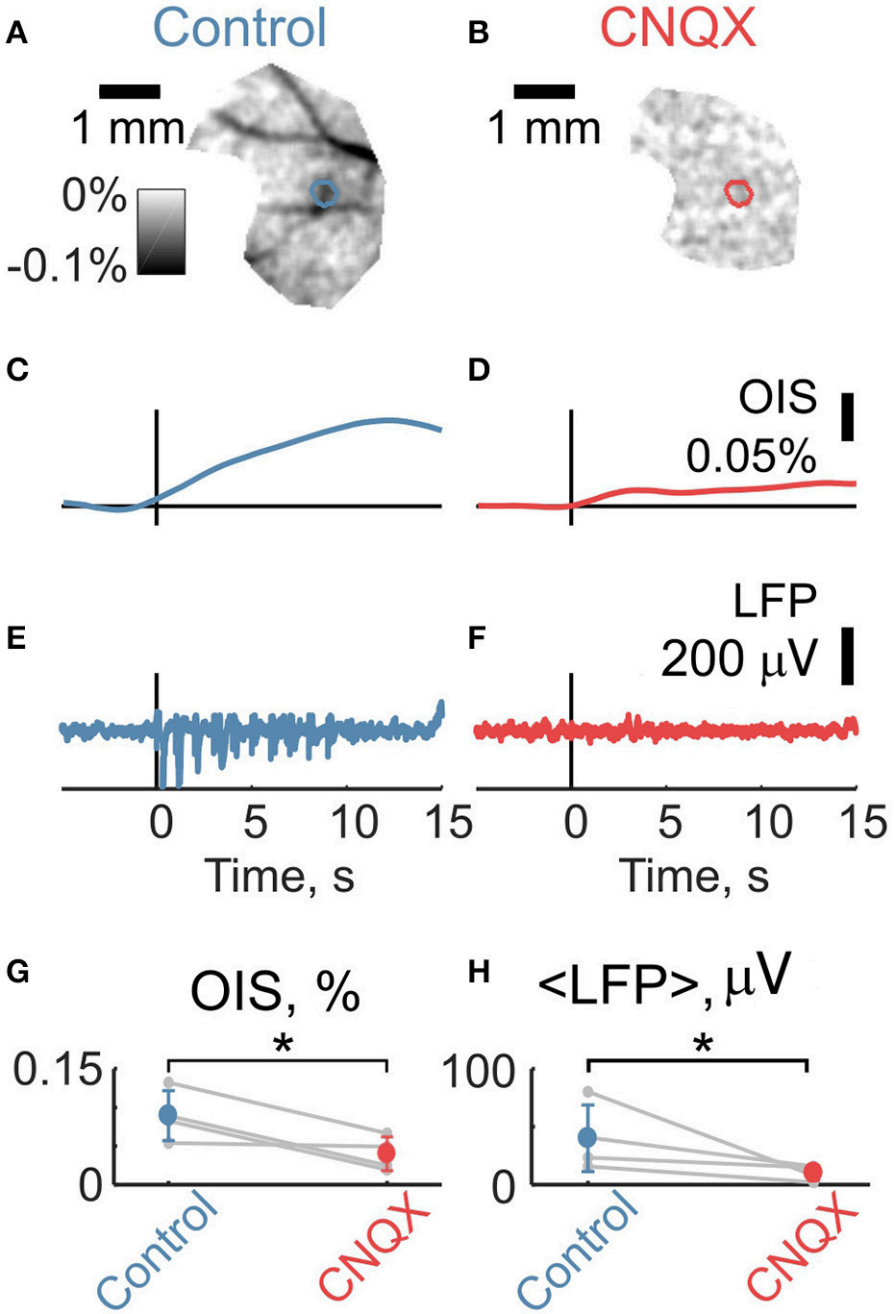
Intracortical application of CNQX suppresses sensory-evoked LFP and OIS. **(A,B)** Examples of OIS maps recorded before and after CNQX injection in a P6 rat. **(C,D)** OIS time courses averaged from OIS masks from **(A,B)**. **(E,F)** LFP time courses recorded using glass pipettes (2-6 MOhm) before and after CNQX injection correspondingly to **(A,B)**. **(G,H)** Group statistics for OIS amplitudes and average LFP for a P5-7 group of *N*_*rats*_ = 4. Circle markers represent a group average and error lines—a group standard deviation. The asterisk (^*^) represents significant changes in group values before and after CNQX injection (Mann-Whitney U-test, *P* < 0.05).

We then compared OIS amplitudes and average LFP in the P5-7 group. In spite of the fact that both OIS amplitude and the average LFP exhibited nonlinear dependence on stimulation rate (Figures 6A,C), OIS amplitude highly correlated with the average LFP in all three experiments (Figure 6B) with a Pearson correlation coefficient of 0.8 ± 0.1. In addition, after injection of the AMPA/kainate receptor antagonist, CNQX, into the principle barrel using a glass pipette (2–6 MΩ), we observed a significant decrease in both average LFP, from 40±28 μV/s to 10±6 μV/s, and OIS amplitude, from 0.09±0.03% to 0.04±0.02% (*N*_*rats*_ = 4, P5-P7, Mann-Whitney U-test, *P* < 0.05) (Figures 7G,H). We may therefore conclude, that the early scattering component in the neonatal barrel cortex, as recorded in the RED spectrum, critically depends on the sensory-evoked synaptic activity.

## 4. Discussion

### 4.1. OIS decomposition into HR and LS

Multispectral OIS imaging was shown to reveal the parameters of HR using the modified Beer-Lambert law (MBLL) (Delpy et al., 1988; Kocsis et al., 2006; Ma et al., 2016). To qualitatively assess LS we introduced a scattering pseudo-chromophore similarly to Kohl et al. (2000). The resulting model used in this work, therefore, involves two chromophores associated with hemoglobin (HbR, HbO) and one scattering pseudo-chromophore (S):

(1) $$\begin{array}{r} OIS=\Delta \mu_{a}D_{a}+\mu_{s}^{\prime }\Delta SD_{s} \end{array}$$

where μ_*a*_ = [*HbO*]ϵ_*HbO*_ + [*HbR*]ϵ_*HbR*_, ϵ – extinction coefficient, $\mu_{s}^{\prime }$ – reduced scattering coefficient, *D*_*a*_ and *D*_*s*_ – differential pathlenghts for absorbance and scattering respectively.

To account for the illumination correction during preprocessing we first, assumed that reference and OIS regions share similar core optical properties and second, that the sensory-evoked changes are small. Therefore the subtraction of reference intensity in the illumination correction *I*(*t*) − *I*_*ref*_(*t*) contrasts only the barrel-specific changes. The appropriate measure for a light path in the contrasted region length was chosen to equal to a doubled L4 depth *d*_*IV*_.

(2) $$\begin{array}{r} OIS\approx \left(\Delta \left[HbO\right]\epsilon_{HbO}+\Delta \left[HbR\right]\epsilon_{HbR}+\mu_{s}^{\prime }\Delta S\right)\cdot 2d_{IV} \end{array}$$

The exact values for $\mu_{s}^{\prime }$ were taken from Ma et al. (2016), van der Zee et al. (1993), *d*_*IV*_ – from Mitrukhina et al. (2015) for P7 rats (0.35 mm) and from Paxinos and Watson (1997) for adult rats (0.75 mm). A complete set of model parameters is shown in Table S2.

**Figure 8:**
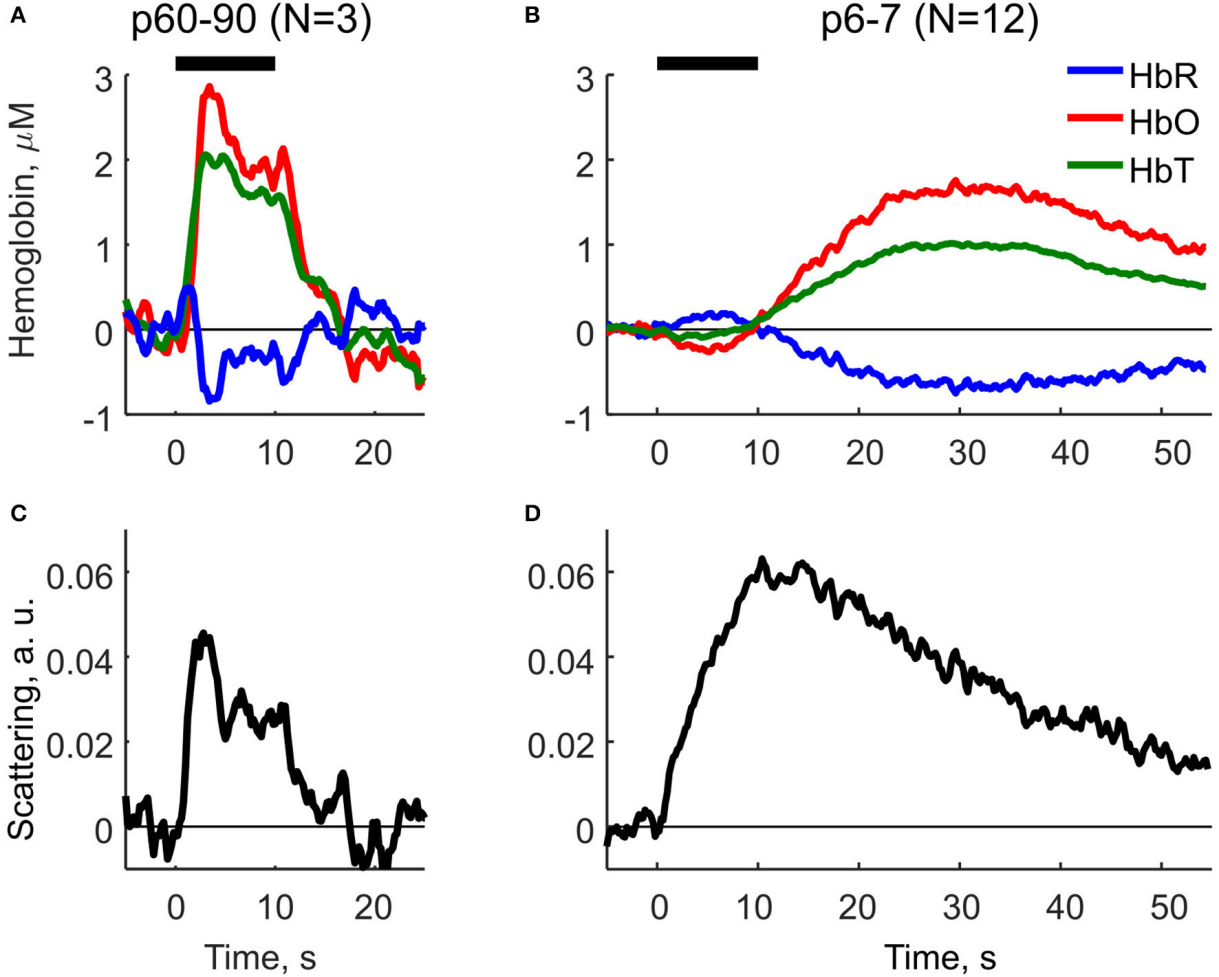
Decomposition of OIS into the hemodynamic response and the light scattering using the modified Beer-Lambert Law (Equation 2) for adult (P60-90) and neonatal (P6-7) rats. **(A,B)** Parameters of the hemodynamic response in adult and neonatal rats: oxyhemoglobin (HbO), deoxyhemoglobin (HbR) and total hemoglobin (HbT). **(C,D)** Scattering component in adult and neonatal rats as calculated using MBLL. A solid horizontal line represents tactile stimulation period.

Using Equation (2) we compared the parameters of HR and LS in neonatal (P6-7) and adult (P60-90) rats (Figure 2). In adult rats, HR and LS exhibited a characteristic profile consistent with the findings in the adult visual cortex (Malonek and Grinvald, 1996). The components were characterized by a rapid increase in HbO and HbT, a decrease in HbR (Figure 8A) and an increase in LS (Figure 8B) starting shortly after stimulation onset. Both HR and LS components were localized within the 10 s stimulation period and largely overlapped. The decrease in HbR was preceded by a short increase at the very onset of sensory stimulation—the initial dip as observed in Malonek and Grinvald (1996), Sheth et al. (2003). Neonatal rats exhibited a different dynamic profile of HR and LS components (Figures 8C,D). Firstly, the dynamics of both components were much slower in neonates than in adults. Secondly, HR and LS components were clearly separated in time. The neonatal sensory-evoked OIS could be split into two phases, an early phase dominated by LS and a late phase dominated by HR. The early phase was localized within the 10s stimulation period and was characterized by an increase in LS and a mild negative HR with a small increase in HbR and a decrease in HbO. Most importantly, total hemoglobin concentration (HbT) remained unchanged during the early phase. *We must admit, though, that the early negative HR is small, and, its detailed description requires additional studies with higher resolution equipment*. The second phase of the optical signal started at the end of the stimulation period and was characterized by a positive HR including an increase in HbT, HbO and a decrease in HbR, which attained their maximal values at approximately 30 s after the onset of stimulation. The late increase in HbT, which is known to correlate with CBF (Grubb et al., 1974), evidences that the sensory-evoked hyperemia in neonatal rats is considerably delayed compared to in adults. LS in neonates peaked at the end of the stimulation period (10 s) and declined monotonically during the late phase. The half-amplitude recovery period of LS, the time span required for a signal to lower by 0.5 of its amplitude equaled *T*_0.5_ ≈ 25 s. Based on these calculations, we conclude, that in neonatal rats the functional hyperemic response is significantly delayed from the sensory-evoked neuronal activity and that the early phase of OIS is dominated by LS.

### 4.2. Light scattering response in the neonatal rats

Our results indicate that the early phase of the sensory-evoked OIS response in the neonatal rat barrel cortex is dominated by LS. Changes in LS primarily involved an increase in brain tissue transparency *in vivo* (Figure 4). Previously, LS properties had been studied mostly *in vitro* using brain slices, where HR-associated effects are absent. It was shown, that the scattering signal in the active tissue is caused by the transmembrane ionic flux resulting in cellular osmotic stress with subsequent swelling of neurons and glial cells (MacVicar and Hochman, 1991; Aitken et al., 1999; Macvicar et al., 2002). However, in the intact brain *in vivo*, contribution to the optical signals from LS is far less prominent because of the dominating HR, so special conditions or special techniques are required to reveal LS. In the mouse olfactory bulb, OISs arise from activity-dependent swelling of sensory neuron axons (Vincis et al., 2015). Also, the first ~100 ms of OIS in the adult rat somatosensory cortex is primarily generated by LS (Rector et al., 2005). Even if the LS and HR components overlap in time such as in the cat visual cortex, the LS contribution can be extracted by a simple model of spectral decomposition (Malonek and Grinvald, 1996). Yet, it remains questionable how well the model decouples hemodynamics and scattering. This question could be resolved by using more complex physical models of light propagation in biological tissues (Delpy et al., 1988; Kohl et al., 2000; Kocsis et al., 2006) as well as by direct Monte Carlo simulation of light propagation in biological tissue (Wang et al., 1995; Fang and Boas, 2009). Our data on the direct light transmission measurements (Figure 4) supported by the decomposition of OIS components (Figure 8) suggest that the neonatal rat model is one of few models unambiguously exhibiting LS as a major component of optical signals. This uniqueness is governed by temporal separation of LS and the late HR.

The LS component of the sensory-evoked OIS in the neonatal rat barrel cortex may involve the same mechanisms as in adults such as cellular swelling, with some developmental differences. We found that LS raises and decays much slower than in adults (Figure 8). LS was suppressed by an intracortical injection of the AMPA/kainate glutamate receptor antagonist CNQX (Figure 7), which also strongly reduced electrical responses in keeping with previous observations (Minlebaev et al., 2007; Colonnese et al., 2010). These results are different from the findings in the mouse olfactory bulb, where suppression of synaptic transmission barely affected LS response and where the optical signals were primarily generated by swelling of the axonal afferents (Vincis et al., 2015). It is plausible that the CNQX-sensitive LS in the neonatal barrel cortex is due to the cellular swelling caused by the transmembrane ion influx during synaptic activation of the glutamate and GABA activated ion channels and postsynaptic spikes. The relatively small number of synaptic connections at these early stages of synaptogenesis may account for the slow rise time of the LS signals (Valiullina et al., 2016). The slow decay of LS may be due to a slow restoration of the electrolytic and water homeostasis in the neonatal tissue. This may involve relatively weak expression and function of ionic cotransporters such as KCC2 (Watanabe et al., 2015), and also the reduction in Na,P-ATPase activity during the early phase of the response caused by transient hypoxia (evidenced by HbO reduction) and the probable associated fall in intracellular ATP.

### 4.3. Delayed HR in the barrel cortex of neonatal rats

We showed that a positive HR in neonatal rats was considerably delayed from the stimulus onset (Figure 8). Our findings are consistent with slower and weaker HR shown in fMRI studies of preterm human infants (Arichi et al., 2012; Allievi et al., 2016). This effect could arise from poor cerebral vascular autoregulation in newborn rats. The neurovascular unit (NVU) which is responsible for the neurogenic regulation of CBF comprises neurons and astrocytes on one side and arterial smooth muscles and endothelium on the other (Attwell et al., 2010). The role of the capillary level regulation of CBF *in vivo* is arguable (Hill et al., 2015), though pericytes were shown to modulate capillary lumen *in vitro* (Hall et al., 2014). Throughout its development the NVU functions undergo continuous adjustments supported by extensive neuro-, glio- and angiogenesis (Harris et al., 2011). Neonatal cerebral vasculature volume is 2–4 times lower than in adults (Keep and Jones, 1990; Risser et al., 2009), arterial sprouting is poor (Norman and O'Kusky, 1986) and the number of chemical synapses are extremely low early in development (Valiullina et al., 2016). Each of the NVU constituents may contribute to the neonatal HR delay, and exhaustive quantitative modeling is required to decipher the true nature of this delay. Yet, recent computational studies show that such modeling is feasible (Buxton et al., 2004; Zheng et al., 2005; Jolivet et al., 2015). The less obvious explanation for the HR delay may arise from the effect of a sensory-evoked decrease in CBF observed in neonatal rats when subjected to strong stimulation (Zehendner et al., 2013). Our data reveal that in neonatal rats light transmittance in the active region is increased which may indicate an excess of osmotic stress in the tissue. Coupled with poor cerebral vascular autoregulation in neonates, both facts may evidence a local ischemic condition—mechanical compression of the adjacent vasculature and local CBF decrease—which, in turn, could account for the HR delay.

### 4.4. OIS limitations for the neuroimaging in very young neonates

We have found that OIS could be consistently recorded from the barrel cortex of neonatal rats starting from age P4 but not before. This is despite the formation of thalamocortical connections already at birth and the functional segregation of barrels by P3 (Mitrukhina et al., 2015). The absence of a detectable OIS before P4 could be explained by the technical limitations of our experimental setup and secondly, by the physiological aspects of developing rats. Indeed, we found that the amplitude of OIS positively correlates with age. Therefore OIS could exist even before P3, with the signal amplitude below the detection threshold of the equipment used in the present study. The reasons for weaker OIS in the neonatal rats have been discussed above. Use-dependent depression of synaptic transmission which is particularly robust in the most immature animals may contribute to this phenomenon too. Although highly hypothetical, one could also add the lack of the feedforward perisomatic GABAergic synapses in L4 neurons until the age P4 (Daw et al., 2007; Minlebaev et al., 2011). Limited activation of the GABAergic synapses in very young animals would result in a much smaller transmembrane chloride influx and smaller swelling during the sensory-evoked responses. Another explanation could be that chloride currents are outwardly directed in keeping with the depolarizing GABA hypothesis, that would counterbalance the intracellular osmotic changes. However, recent *in vivo* findings question this hypothesis (Kirmse et al., 2010; Valeeva et al., 2013).

## 5. Conclusion

We performed multispectral optical intrinsic signal imaging of sensory-evoked responses in the rat barrel cortex starting from age P4 onwards. Applying the MBLL, we found that the optical signal in neonatal rats is organized into two temporally separated processes: an early phase with a change in tissue light scattering (LS) and a late phase with a hemodynamic response (HR). We also found that the HR delay shortens with age and largely overlaps with LS by the end of the first postnatal month. These findings may be of great importance for optical functional neuroimaging in human infants, particularly in preterm babies.

## Author contributions

MM, RK: Study concept and supervision. MS, DS: Acquisition of data and analysis. MS: Drafting of the manuscript. RK: Critical revision of the manuscript.

### Conflict of interest statement

The authors declare that the research was conducted in the absence of any commercial or financial relationships that could be construed as a potential conflict of interest.

## Supplementary material

Supplementary Video 1 Frame by frame comparison of OIS recorded in a P6 rat using IR reflected, and IR transmitted light. Dashed regions in OIS maps indicate regions corresponding to the D1 barrel. Below are the trial-averaged OIS time courses averaged in the D1 regions (*N_trials_* = 20). The black horizontal line depicts stimulation interval. Note that OISs are inverted (upwardly-directed signals correspond to darkening and downwardly-directed signals correspond to brightening).

Figure S1 **(A)** Hemoglobin exctinction coefficient (Zijlstra et al., 1994). **(B)** Exact diode spectra diodes spectra from Table S1 measured with Thorlabs CCS175 spectrometer.

Figure S2 Optimal stimulation studies for different age groups to maximize OIS. **(A–C)** are group statistics for OIS amplitude for P6–7, P11–14, and P15–18 group. **(D)** Dependency of optimal stimulation rates on the animal age calculated as a group center age. Markers represent average and whiskers—standard deviation of OIS amplitudes in a group. Solid markers represent groups statistics with N_*rats*_ ≥ 3 and hollow markers–otherwise. Pearson correlation coefficient between stimulation rates and ages appeared to be 0.65 (dashed line for illustration).

Table S1 Diodes for multispectral OISi, their wavelength of maximal emission (λ_max_) and averaged extinction coefficient of oxy- and deoxy-hemoglobin (ϵ_HbO_ and ϵ_HbR_). Calculations are based on Zijlstra et al. (1994) and diodes spectra measurement using Thorlabs CCS175 spectrometer.

Table S2 Parameters values used for MBLL to decompose OIS signals.ϵ_nap_ stands for the extinction coefficient corrected for the Napierian base, $\mu_{s}^{\prime }$–for the reduced scattering coefficient.
