# Supplementary material for: Developmental Changes in Sensory-Evoked Optical Intrinsic Signals in the Rat Barrel Cortex
Source: Front Cell Neurosci. 2017 Dec 12;11:392. doi: 10.3389/fncel.2017.00392 (PMC5733043; doi:10.3389/fncel.2017.00392)
Supplement: Figure S1 — (A) Hemoglobin exctinction coefficient (Zijlstra et al., 1994). (B) Exact diode spectra diodes spectra from Table S1 measured with Thorlabs CCS175 spectrometer. [file DataSheet1.PDF]

Supplementary Material:
Optical intrinsic signal imaging of delayed hemodynamic response and tissue transparency in the barrel cortex of rats early in development

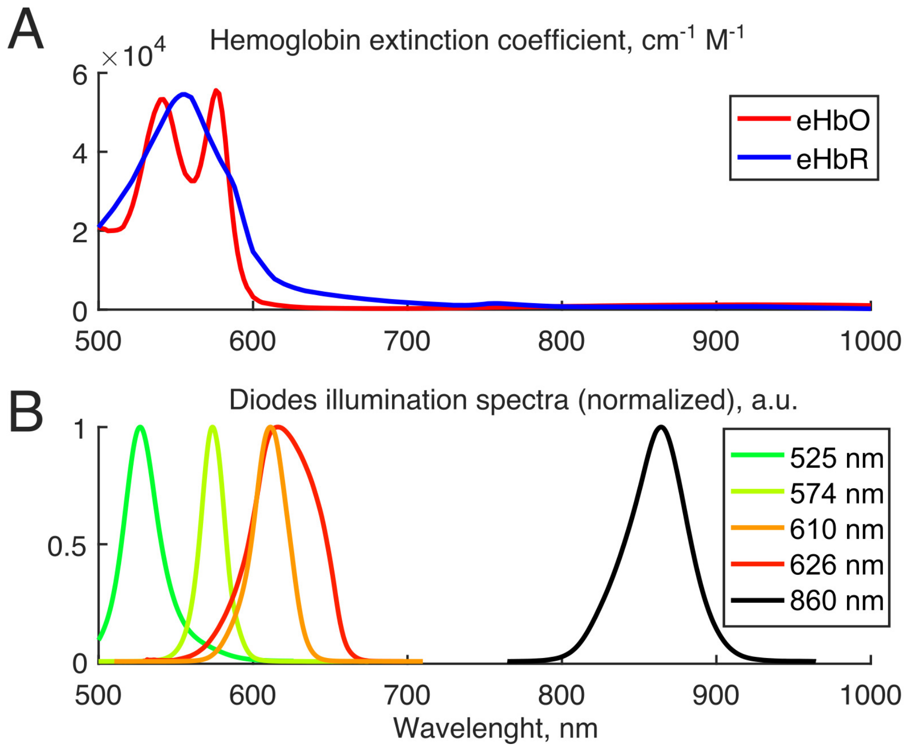

Figure S1: (A) Hemoglobin exctinction coefficient (Zijlstra et al., 1994). (B) Exact diode spectra diodes spectra from Tab.S1 measured with Thorlabs CCS175 spectrometer.

Table S1. Diodes for multispectral OISi, their wavelength of maximal emission (λ_{max}) and averaged extinction coefficient of oxy- and deoxyhemoglobin (ϵHbO and ϵHbR). Calculations are based on (Zijlstra et al., 1994) and diodes spectra measurement using Thorlabs CCS175 spectrometer.

Spectrum notation	Diode name	λ_{max}, nm	ϵHbO $cm^{-1}M^{-1}$	ϵHbR $cm^{-1}M^{-1}$
GREEN	Arlight ARPL GREEN 3W	525	34300	36600
GREEN	Kingbright L-53MGC (L-7113MGC)	574	43900	41600
RED	Kingbright L-53SET (L-7113SET)	610	3900	11600
RED	Arlight ARPL RED 3W + Rosco SG86 E703 E5455	626	3700	10100
IR	Kingbright L-53SF6C (L-7113SF6C)	860	800	400

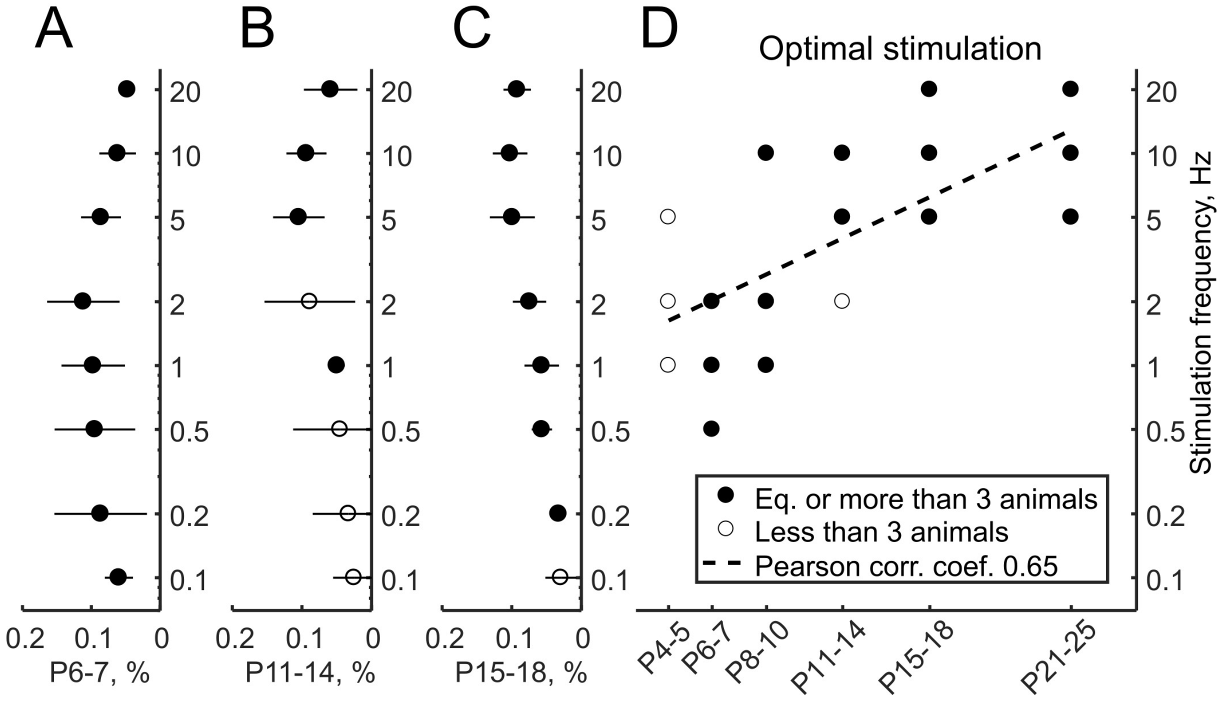

Figure S2: Optimal stimulation studies for different age groups to maximize OIS. (A), (B) and (C) are group statistics for OIS amplitude for P6-7, P11-14 and P15-18 group. (D) Dependency of optimal stimulation rates on the animal age calculated as a group center age. Markers represent average and whiskers – standard deviation of OIS amplitudes in a group. Solid markers represent groups statistics with $N_{rats} \geq 3$ and hollow markers – otherwise. Pearson correlation coefficient between stimulation rates and ages appeared to be 0.65 (dashed line for illustration).

Table S2. Parameters values used for MBLL to decompose OIS signals. ϵ_{nap} stands for the extinction coefficient corrected for the Napierian base, μ_s' – for the reduced scattering coefficient

Spectrum notation	$\epsilon_{nap}HbO$ $mm^{-1}\mu M^{-1}$	$\epsilon_{nap}HbR$ $mm^{-1}\mu M^{-1}$	μ_s', mm^{-1}
GREEN	0.0089	0.0090	3.5
RED	0.0009	0.0025	3.2
IR	0.0002	0.0002	2.2
